# Flexible Two-Phase studies for rare exposures: Feasibility, planning and efficiency issues of a new variant

**DOI:** 10.1186/1742-5573-5-4

**Published:** 2008-10-01

**Authors:** Pascal Wild, Nadine Andrieu, Alisa M Goldstein, Walter Schill

**Affiliations:** 1 INRS, French National Institute for Research and Safety, Department of Epidemiology, France; 2 INSERM, U900, Paris, F-75248, France; 3 Institut Curie, Paris, F-75248, France; 4 Ecole des Mines de Paris, ParisTech, Fontainebleau, F-77300, France; 5 Genetic Epidemiology Branch, Division of Cancer Epidemiology and Genetics, National Cancer Institute, NIH, DHHS, USA; 6 BIPS, Bremen Institute for Prevention Research and Social Medicine, University of Bremen, Germany

## Abstract

The two-phase design consists of an initial (Phase One) study with known disease status and inexpensive covariate information. Within this initial study one selects a subsample on which to collect detailed covariate data. Two-phase studies have been shown to be efficient compared to standard case-control designs. However, potential problems arise if one cannot assure minimum sample sizes in the rarest categories or if recontact of subjects is difficult.

In the case of a rare exposure with an inexpensive proxy, the authors propose the flexible two-phase design for which there is a single time of contact, at which a decision about full covariate ascertainment is made based on the proxy. Subjects are screened until the desired numbers of cases and controls have been selected for full data collection. Strategies for optimizing the cost/efficiency of this design and corresponding software are presented. The design is applied to two examples from occupational and genetic epidemiology. By ensuring minimum numbers for the rarest disease-covariate combination(s), we obtain considerable efficiency gains over standard two-phase studies with an improved practical feasibility.

The flexible two-phase design may be the design of choice in the case of well targeted studies of the effect of rare exposures with an inexpensive proxy.

## Introduction

For rare exposures, the power of epidemiological studies depends mainly on the rarest disease-exposure combinations. For example, in population-based case-control studies the limiting factor is frequently the number of exposed cases and/or controls. One approach that may substantially increase power for these types of studies is the two-phase study design.

The two-phase design [1-4] consists of an initial (Phase One) large study with known disease status and easily collectible or inexpensive covariate information. Within this initial study one selects a subsample on which to collect detailed covariate data (Phase Two). In Phase Two, one may deliberately oversample the subjects with the rarest exposure-disease combinations based on the available Phase One information, consequently increasing power. Appropriate statistical methods [5] correct for the biased sampling by incorporating the statistical distribution of the available information among cases and controls from Phase One. The data collection of Phase Two usually proceeds in one of two ways. The first approach includes recontacting selected study subjects from Phase One to obtain detailed covariate information. However, with secondary data collection, potential problems may arise if recontacting subjects is difficult, if cases have died, or if response rates are low. Alternatively, one may collect full raw data at first contact for all participants and process only selected subjects. An example would be a molecular or genetic epidemiologic study in which biological specimens were obtained for all cases and controls but only a subsample were genotyped (see [6] for another example). This may, however, be considered wasteful since only a fraction of the collected data is used.

As an alternative, we propose a new variant of the two-phase design called the flexible two-phase design, for which there is a single time of contact. Phase One data are collected for all subjects and Phase Two subjects are selected for immediate complete data collection based on their basic Phase One information. The key principle of this new variant is to fix a priori stratum-wise numbers of cases and controls for full data collection and recruit Phase One subjects until the required numbers of subjects in each stratum are reached.

We describe the proposed study design and its implementation in terms of power, cost/efficiency considerations and statistical analysis. We illustrate its applicability using two examples from occupational and molecular/genetic epidemiology.

## Steps in the planning and the analysis of flexible two-phase studies

We start by defining several key variables and then describe the proposed set-up for the study design. First, define Z, a discrete proxy variable for the exposure(s) of interest (X). Z needs to be collected and available at Phase One. Then, compute the power for several design options within the flexible two-phase design (see below). Based on these computations, select the design option which produces the best compromise between power and feasibility in terms of subject availability, cost and other study-specific criteria that will permit achievement of the study aims.

The four major steps for the set-up of a study with the proposed design are as follows:

### Design set-up

1. Identify a stratification variable Z which is an easily available proxy of the exposure(s) of interest X. The number of strata (J) will equal the number of response choices for Z.

2. For each stratum, fix the number of cases and controls (n_ij_), based on study power and cost considerations, for whom the exposure of interest X and covariates will be assessed. From n_ij_, compute their expected distributions according to X and the numbers of cases and controls who will need to be screened at Phase One.

### Data collection

3. Screen subjects for Z and keep cases and controls for full data collection (i.e. the variable(s) of interest X and potential confounders) until the numbers of cases and controls fixed in step 2 are reached.

4. Within each stratum j, count the number of cases and controls that were screened in Phase One at Step 3.

### Computation of expected numbers and power

As mentioned above, the expected Phase One numbers depend on the fixed stratum-specific Phase Two numbers. They also depend on the study hypotheses including exposure prevalences and odds ratios. Other assumptions, common to all types of two-phase studies, quantify how well the Phase One strata predict the exposure of interest (sensitivity and specificity of proxy Z). The formulas for expected Phase One and Phase Two numbers are given in Appendix 1. From these numbers, one can compute, using specific variance computations given in Schill and Drescher [5], the expected asymptotic variance and the statistical power. A corresponding STATA (StataCorp, College Station Texas) program for data analysis and power computations is included as an online add-on to this paper.

### Planning options

A critical issue is how to optimize, in terms of cost and power, the fixed stratum-wise numbers of cases and controls with full data collection. This complex problem has been addressed in different contexts [7-10]. However, one can formulate a general heuristic rule, which has worked well in our applications using Maximum Likelihood as the analysis method. Specifically, choose the numbers of cases and controls for full data collection so that, within both controls and cases, the overall expected Phase Two exposure proportions are as equally distributed as possible. For rare exposures, this means choosing cases and controls to oversample the rarest exposure categories among both groups.

### Statistical analysis

The collected data can be analyzed using any two-phase analysis software. As the second phase sample is a biased sample of the original population, a combined analysis of the Phase One and the Phase Two data relies on weighting of the Phase Two data by the inverse sampling fractions. The two main methods for analysis are maximum likelihood (ML) and weighted likelihood (WL) which differ in the weights used; the more efficient ML estimate iteratively adjusts these weights using the estimated disease model. As such software is not readily available, we included our STATA-based two-phase analysis program "blogit_2P.ado" [see additional file 1]. The software takes as input the disease indicator, the stratum indicator, the Phase One frequencies, the Phase Two frequencies and the independent variables. A help file accessible from within STATA "blogit_2P.hlp" [see additional file 2] is also included as well as an illustrative example [see additional files 3 and 4]. In this paper, we use the ML approach.

## Examples

To demonstrate the potential efficiency of the flexible two-phase approach, we present two examples from occupational and molecular/genetic epidemiology. In the first example, we detail the computations for a given design; in the second, we perform a full search for optimal designs for given scenarios.

### Example 1: Metalworking fluids and bladder cancer

A number of population-based case-control studies have found an association between bladder cancer and metalworking fluids (MWF) exposure (see Calvert [11] for a review). However, because of the low prevalence of the exposure, the numbers of exposed cases and controls in each study were too small to produce a stable estimate of the association. We use a flexible two-phase study to illustrate the efficiency gain over a standard case-control study, considering as a proxy of MWF exposure "having worked in the metal industry". In practice, when contacting cases and controls, for instance in a telephone interview, one of the first questions to the volunteers would be: "Have you ever worked in the metal industry?". Based on the answer to this question the subject would then be included (or not) in Phase Two; that is, the interview would be continued to assess a detailed work history and confounder information.

Table 1 details the assumptions. The study proceeds along the four steps as follows:

**Table 1:** Scenario for Example 1

**Variables and parameters characterizing the set-up**	**Values of parameters and variables**
Stratification/Proxy Z (with J strata)	Past work in metal industry
	No: Z = 1
	Yes: Z = 2

Phase One prevalence among controls (τ^0^_j_)	Z = 1: τ^0^_1 _= 80%
	Z = 2: τ^0^_2 _= 20%*

Risk factor X (with K outcomes)	Exposure to MWF
	No: X = 1
	Yes: X = 2

Disease Model (Odds Ratios) (ψ_k_)	ψ_1 _= 1: baseline risk
	ψ_2 _= 2^#^

Phase Two prevalence of X among controls by stratum (π^0^_jk_)	Z = 1: π^0^_11 _= 97.5%, π^0^_12 _= 2.5%^&^ Z = 2: π^0^_21 _= 75%, π^0^_22 _= 25%^@^

*20% prevalence of having worked in the metal industry

#Exposure to MWF doubles the risk of bladder cancer

&Among non-metal-industry workers, 2.5% exposed to MWF

@Among metal-industry workers, 25% exposed to MWF

### Study design

1. Stratify subjects by Z (Table 1, Line 1).

2. Per stratum, fix the numbers of cases and controls (160 metal-working and 40 non-metal-working controls, 85 metal-working and 20 non-metal-working cases – Table 2 Column 3) to be included and for whom MWF exposure will be assessed at Phase Two. These numbers were chosen using our heuristic rule to reach 80% power to detect the effect of MWF.

**Table 2:** Design of the flexible two-phase study for Example 1

**Disease Status** (D)	**Metal-workers** Z (*τ*^*i*^_*j*_)	**Fixed number of subjects to be included in Phase Two** (*n*_*ij*_)	**Expected Phase One numbers of subjects to be screened** (*N*_*ij*_)	**Expected Proportion of MWF exposure within strata §** X(*π*^*i*^_*jk*_)	**Expected distribution of subjects by MWF in Phase Two**
			N_0 _= Max(160/20%, 40/80%) = 800		

Control	No (80%*)	40	800*80% = 640	No (97.5%*)	40*97.5% = 39
				Yes (2.5%*)	40*2.5% = 1
	Yes (20%*)	160	800*20% = 160	No (75%*)	160*75% = 120
				Yes (25%*)	160*25% = 40

			N_1 _= Max(85/23.4%, 20/76.6%) = 364		

Case	No (76.6%#)	20	364*76.6% = 278.8	No (95.1%#)	20*95.1% = 19.02
				Yes (4.9%#)	20*4.9% = 0.98
	Yes (23.4%#)	85	364*23.4% = 85	No (60%#)	85*60% = 51
				Yes (40%#)	85*40% = 34

* Values of parameters fixed in Table 1

# Values of parameters computed from parameter values fixed in Table 1 (see Appendix 2)

§ In Phase One controls, the overall expected percentage of MWF exposure is equal to 7%, that is, 2% = 2.5% of 80% non-metal-workers plus 5% = 25% of 20% metal-workers. Similar computations lead to 13% MWF exposure in cases.

### Planned data collection

3. Screen cases and controls until the required numbers in each stratum are reached and assess the detailed exposure to MWF and potential confounders in this sample of 305 subjects.

4. Record the number of subjects screened in order to reach the required sample size. At the planning stage, these numbers are not yet available, but expected numbers can be computed. Assuming 20% metal-workers in the general population, we would expect to screen 800 controls (N_0_) to obtain 160 metal-workers (20% × 800 = 160). Therefore, the number of non-metal worker controls that would have been screened (N_00_) is expected to be 640 (800–160) of which 40 are included in Phase Two for detailed exposure assessment. For the corresponding computations for cases, see Table 2 and Appendix 2.

We note that oversampling the metal-workers has achieved our aim of increased numbers of MWF exposed cases and controls. Among the 200 controls, 41 are exposed (20.5% versus 7% in Phase One) and among the 105 cases, 35 are exposed (33.3% versus 13% in Phase One) (Table 2, Column 6 and Footnote §.

**Figure 1:**
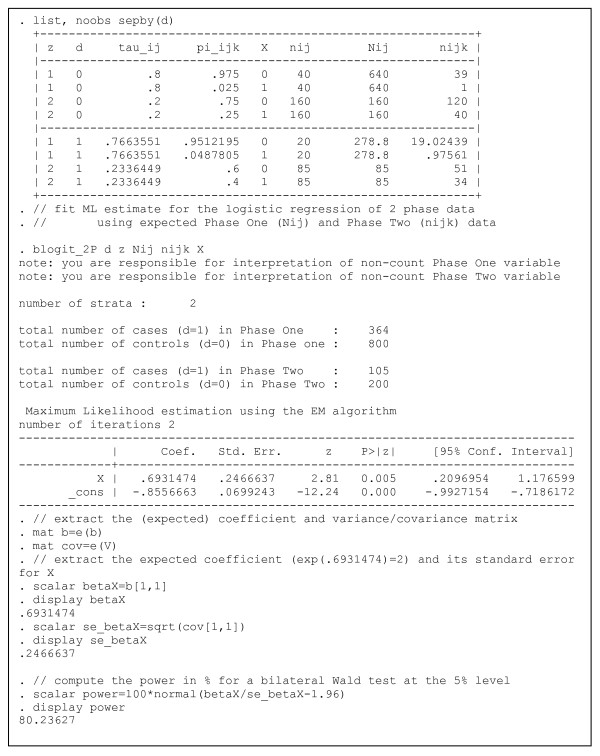
STATA output for Example 1.

Figure 1 shows the STATA output of the analysis of the expected frequencies. The STATA program for this analysis is included as an additional file (figure 1.do [see Additional file 3] using the STATA data file MWF.dta [see Additional file 4] obtained by applying the computations shown in Appendix 2). In this example d, z, X, N_ij_, n_ijk_, respectively denote, the case status (1 = case, 0 = control), the stratum indicator, the metal fluid indicator (X = 1 exposed, X = 0 unexposed), the stratum-wise numbers in Phase One, and the Phase Two numbers by stratum and exposure to metal fluids. The power is computed using a bilateral Wald test at a 5% level using the following formula: Power = Φ(β_x_/se(β_x_)-1.96) = 80.2% where Φ denotes the cumulative standard normal distribution, β_x _the log-odds ratio and se(β_x_) its standard error. The asymptotic standard error se(β_x_) is 0.247 for the log-odds ratio and β_x _= ln(2) = 0.693, as the assumed OR is equal to 2. In contrast, a standard case-control study, in which 200 controls and 105 cases were randomly selected, would yield a se(β_x_) = 0.400, corresponding to 40.9% power using the same formula.

### Example 2: Detection of gene-environment interaction

Molecular/genetic epidemiology studies identify genes involved in disease risk, estimate the strength of the disease-gene association and investigate modifier factors that may interact with the susceptibility genes. The study of interactions between genes and "environmental" factors is often challenging because of the rarity of having both factors, i.e., being exposed to the environmental factor of interest and carrying a deleterious allele.

We present a search for an optimized flexible Two-Phase design, in this setting, assuming that an inexpensive proxy of the deleterious allele (e.g., family history of disease) is available.

#### The scenarios

We consider a rare deleterious allele G with 1% prevalence (PG), interacting with an environmental exposure E with 20% prevalence (PE). The odds ratios for E, G and their interaction (I) are respectively 2, 3 and 5 (Table 3).

**Table 3:** Scenarios for Example 2

**Variables and parameters required for set-up**	**Formulas and values of parameters**
Stratification/Proxy Z (with J strata)	Environmental exposure E and Gene proxy S_G_
	J = 4
	Z = 1: E^- ^S_G_^-^, Z = 2: E^- ^S_G_^+^, Z = 3: E^+ ^S_G_^-^, Z = 4: E^+ ^S_G_^+^

Phase One prevalence among controls (τ^0^_j_):	τ^0^_1 _= Pr(E^-^)Pr(S_G_^-^) = (1 - P_E_)[(1-Se)P_G_+Sp(1-P_G_)]
P_E _= 20%	τ^0^_2 _= Pr(E^-^)Pr(S_G_^+^) = (1 - P_E_)[SeP_G_+(1-Sp).(1-P_G_)]
P_G _= 1%	τ^0^_3 _= Pr(E^+^)Pr(S_G_^-^) = P_E_[(1-Se)P_G_+Sp(1-P_G_)]
	τ^0^_4 _= Pr(E^+^)Pr(S_G_^+^) = P_E_[SeP_G_+(1-Sp).(1-P_G_)]

Risk factor X (with K outcomes)	Exposure to E and exposure to G: K = 4
	X = 1: E^- ^G^-^, X = 2: E^- ^G^+^, X = 3: E^+ ^G^-^, X = 4: E^+ ^G^+^

Disease Model (Odds Ratios ψ_k_)	ψ_1 _= 1, ψ_2 _= 3, ψ_3 _= 2, ψ_4 _= ψ_2 _× ψ_3 _× OR_I _= 30

Phase Two prevalence of X among controls by stratum (π^0^_jk_)	Z = 1: π^0^_11 _= (1 -P_E_)Sp(1-P_G_)/Pr(S_G_^-^),
	π^0^_12 _= 1 - π^0^_11_, π^0^_13 _= π^0^_14 _= 0
	Z = 2: π^0^_21 _= (1 - P_E_)(1 - Sp)(1-P_G_)/Pr(S_G_^+^),
	π^0^_22 _= 1 - π^0^_21_, π^0^_23 _= π^0^_24 _= 0
	Z = 3: π^0^_31 _= π^0^_32 _= 0, π^0^_33 _= P_E _Sp(1-P_G_)/Pr(S_G_^-^),
	π^0^_34 _= 1 - π^0^_33_
	Z = 4: π^0^_41 _= = π^0^_42 _= 0, π^0^_43 _= P_E _(1 - Sp)(1-P_G_)/Pr(S_G_^+^),
	π^0^_44 _= 1 - π^0^_43_

*Se = sensitivity; Sp = specificity

We further assume that the proxy of the susceptibility gene (SG) and the environmental exposure (E) are available at Phase One for an unlimited number of controls. However, we restrict the number of cases available in Phase One to a maximum of 2000 cases. We further assume that capacities for genotyping restrict the total number of subjects (cases + controls) that can be included in Phase Two to a maximum of 1200 subjects. We assume that the cost of genotyping is 20 times the cost of screening. Such a cost ratio would arise if, for example, a SNP array costs $100 and 15 minutes for a trained interviewer screening a subject for E and SG costs $5. We repeat the design search for each combination of sensitivity (Se) and specificity (Sp) of 0.6, 0.7, 0.8, and 0.9.

#### Planning the design

The aim of the flexible two-phase approach is to choose subjects for genotyping to optimize the study power for given costs. This is achieved by oversampling subjects with positive gene proxy and environmental exposure.

In practice, such oversampling could be done during case/control recruitment using a short interview that allows assessment of the environmental exposure and the gene proxy (e.g., a family history of disease) and getting a blood/buccal sample (for genotyping) only for the subjects sampled for Phase Two based on the results of this first interview.

Step 1: The stratification is by gene surrogate and environmental exposure (Table 3, line 1).

Step 2 entails choosing the stratum-wise numbers of cases and controls to be included in Phase Two. We use our general heuristic rule with respect to E and fix at 50% the target numbers of E+ and E- to be included in Phase Two among cases and controls. The amount by which we oversample SG+ will be considered through use of two additional parameters, the proportion of controls ρ0 with SG+ and the proportion ρ1 of cases with SG+. For example, if we selected 800 controls and 400 cases with proportions ρ0 = 80% and ρ1 = 60%, this would correspond to 800*50%*80% = 320 E+ SG+ controls, 400*50%*60% = 120 E+ SG+ cases, 800*50%*20% = 80 E+ SG- controls and so on.

#### Comparing designs

We now consider a series of design options for this example for which we compare power and cost. To meet the constraints on availability and capacity fixed above, the designs considered have numbers of cases ranging from 100 to 600 and numbers of controls from 400 to 1100 in steps of 100, with a maximum of 1200 subjects to be included in Phase Two. For each of these combinations, ρ0 and ρ1 are varied from 40% to 90%. This corresponds to several hundred possible designs for each combination of sensitivity and specificity of SG.

Table 4 shows, for each combination of sensitivity and specificity, the design which achieves the maximal power to detect OR_I _= 5. Only designs achieving 80% power are shown. For example, if SG has 80% specificity and 70% sensitivity, the design with the highest power would include 400 cases and 800 controls with ρ_1 _= ρ_0 _= 90% SG+ (Table 4, line 4). We would, thus, include 90%*400 = 360 SG+ cases and 720 SG+ controls for genotyping. The expected numbers of cases to be screened would be 1889 and the expected number of controls would be 8780.

**Table 4:** Designs with maximal power of detecting the interaction, according to sensitivity and specificity

Gene-surrogate		Flexible two-phase design options				Expected Phase One counts		Power#	Cost*
Spec	Sens	n_0_	n_1_	ρ_0_†	ρ_1_‡	N_0_	N_1_		

70%	80%	800	400	90%	90%	5902	1373	83%	1564
70%	90%	800	400	90%	90%	5882	1325	87%	1560
80%	60%	800	400	90%	90%	8824	1988	87%	1741
80%	70%	800	400	90%	90%	8780	1889	91%	1733
80%	80%	800	400	90%	90%	8738	1800	94%	1727
80%	90%	800	400	90%	90%	8696	1718	96%	1720
90%	60%	900	300	90%	90%	19286	2000	98%	2264
90%	70%	900	300	90%	90%	19104	2000	99%	2255
90%	80%	900	300	90%	90%	18925	1960	99.6%	2244
90%	90%	900	300	90%	90%	18750	1835	99.8%	2229

Table 5 shows, for each combination of sensitivity and specificity, the design which achieves the minimal cost with 80% power to detect OR_I _= 5. Using the same example as above, this design would include 300 cases with 80% SG+ and 600 controls with 90% SG+. This would imply screening 1259 cases and 6585 controls and would correspond to a 25% cost decrease compared to the most powerful design (1292 vs. 1733) (Table 5, line 4).

**Table 5:** Designs with minimum cost among designs with 80% power of detecting the interaction

Gene-surrogate		Flexible two-phase design options				Expected Phase One counts		Power#	Cost*
Spec	Sens	n_0_	n_1_	ρ_0_†	ρ_1_‡	N_0_	N_1_		

70%	80%	700	500	90%	80%	5163	1525	81%	1534
70%	90%	600	500	90%	80%	4412	1472	80%	1394
80%	60%	700	300	90%	90%	7721	1491	80%	1461
80%	70%	600	300	90%	80%	6585	1259	80%	1292
80%	80%	500	300	90%	80%	5461	1200	80%	1133
80%	90%	400	400	90%	80%	4348	1528	81%	1094
90%	60%	400	400	70%	50%	6667	1683	81%	1217
90%	70%	500	300	50%	50%	5896	1169	80%	1153
90%	80%	500	300	40%	60%	4673	1307	80%	1099
90%	90%	500	300	40%	50%	4630	1019	82%	1082

# Analysis approach: Maximum likelihood

* the study cost is computed as the sum of the number of screened subjects divided by 20 plus the number of subjects included in Phase Two.

† ρ_0 _is the proportion of S_G_^+ ^controls included in Phase Two

‡ ρ_1 _is the proportion of S_G_^+ ^cases included in Phase Two

Note that the better the proxy, the more effective the flexible two-phase approach. For example, for a gene proxy with 70% specificity and 80% sensitivity, the most cost effective design costs 1534 units whereas the most cost effective design for a gene proxy with 90% specificity and 90% sensitivity costs 1082 units.

#### Comparison with standard case-control studies

For the scenario considered, the most powerful standard case-control study with 1200 genotyped subjects would include 300 cases and 900 controls with an expected var(β_I_) = 0.96, corresponding to a statistical power of 37%. Achieving 80% power would require var(β_I_) = 0.33. Thus, for a standard case-control study to attain 80% power, it would require genotyping of 870 cases (i.e. 300 × 0.96/0.33) and 2610 controls (i.e. 900 × 0.96/0.33), totaling a cost of 3480 units. This compares to 1534 units in the most cost-effective flexible two-phase design assuming 70% specificity and 80% sensitivity.

#### Comparison with balanced two-phase studies

A second comparison of interest would be a comparison with balanced two-phase studies, the design that is generally recommended in papers on two-phase studies (see [1,2,12]). As mentioned in the introduction, these studies start from a fixed Phase One sample and draw equal numbers in each stratum for Phase Two data collection. In order to be comparable to our flexible design, we considered a design in which 8000 controls and 2000 cases were assessed in Phase One and 800 controls and 400 cases included in Phase Two. As the design is balanced, we selected equal numbers, i.e., 200 controls and 100 cases from each stratum defined by SG × E.

This balanced Two-Phase design is always less efficient than the Flexible Two-Phase design although more efficient than the standard case-control design. For instance, in the preceding example with 70% specificity and 80% sensitivity, the expected variance is var(β_I_) = 0.47, corresponding to a statistical power of 65%. The corresponding cost is 1200+(10000:20) = 1700 units.

## Discussion

Two-phase studies are efficient compared to standard case-control designs. The variant design presented in this paper improves on some aspects of standard two-phase studies. Specifically, with respect to data collection there is only one time of contact. At a time when studies are struggling with decreasing response rates, collection of all necessary data at a single time of contact may result in improved overall participation rates. Moreover, for rare exposures, minimum numbers of exposed subjects can be guaranteed in this design, thus increasing the power, even compared with standard balanced Two-Phase designs. The disadvantage of the flexible two-phase design compared to other designs, including standard two-phase, is the additional complexity in design planning. Another possible disadvantage is that the categories that are relatively easy to fill will be filled quickly during recruitment, while the hard-to-fill categories will take longer to reach their sampling targets. This can produce complex relationships between covariates and recruitment times. This could be alleviated by the randomized recruitment approach proposed by Weinberg and Sandler [13] in which the most common Phase One category would be included in Phase Two with a given probability, chosen so that all categories are filled in at about the same time.

In the examples presented, we focused on rare exposures for which one could identify inexpensive proxies. Using our proposed heuristic rule, this allows oversampling the rare exposure and thus increasing power. This approach is efficient provided the analysis method used is maximum likelihood, thus, implicitly assuming non-differential misclassification, i.e., that the proxy is not a confounder. In practical terms, this means that the disease risk, given exposure, is the same in all strata. If the disease risk varies across strata, the effect of exposure may have to be assessed separately in each stratum resulting in reduced power to detect the effect of exposure in the underrepresented strata.

One major consideration for the flexible two-phase design is the availability of an adequate proxy for Phase One screening. The proxy must be easily obtained on all screened subjects but must also have high sensitivity and specificity. For a study focused on occupational exposures, as in example 1, a question about working in the industry of interest is easily collected and should yield a reasonable proxy for exposure. This binary stratification for the proxy may be extended to increase sensitivity and specificity. For example, one could ask about duration of work in a particular industry, thereby obtaining a proxy of the actual cumulative dose. Similarly, a positive family history was previously shown [14] to be a good proxy for a rare gene with a strong effect. However, as the effect of the allele decreases and its frequency increases (as would be the situation for a low-risk gene) the sensitivity and specificity for family history decreases. In such situations, an alternative proxy for G may need to be considered, such as age at diagnosis, or a quick inexpensive physiologic test during the in-person interview at Phase One. Of course, the more information obtained at Phase One, the more expensive Phase One becomes.

We acknowledge that a gene-environment interaction odds ratio of 5 may be rather extreme for most diseases, particularly given some recent findings, as in [15]. We are currently working on a more topic-oriented comparison of different study designs for detecting gene-environment interactions using a wider range of scenarios and including the Flexible Two-Phase design and case-only design (under the assumption of independence of Genetic and Environmental factors in the population).

In the present paper, we focused on the estimation of a single odds-ratio. However, dose-response estimation is possible, as long as detailed data are available at Phase Two. Similarly, it is possible to adjust for confounders as long as the relevant data are available in Phase Two. However, since the flexible two-phase design is mostly targeted on predefined hypotheses, especially if one oversamples some strata, there may be limited power to test other hypotheses or perform exploratory analyses. For example, exposure to some aromatic amines increases risk for bladder cancer, but this exposure is rare in the metal industry. Thus, the design we considered would have low power for detecting this risk. Many epidemiologic studies are exploratory in that they assess the effects of a large spectrum of factors without focusing on predefined hypotheses. The Flexible Two-Phase design is not adapted to this situation and focuses necessarily on a restricted number of explicitly stated hypotheses. We are, however, convinced that in many circumstances, only studies with predefined hypotheses will allow progress in understanding disease etiology.

## Conclusion

In conclusion, the flexible two-phase design expands the advantages of two-phase designs to substantially increase power for studies of rare disease-exposure combinations. The flexible two-phase design may be the design of choice in well targeted studies of the effect of rare exposures for which inexpensive proxies are available.

## Abbreviations

MWF: metal working fluid; SG: the surrogate of the gene G considered as a risk factor.

## Competing interests

The authors declare that they have no competing interests.

## Authors' contributions

The idea of this new method originated from discussions between PW and WS. PW wrote the first draft, carried out the computations and prepared the tables and figure. NA and AMG contributed the gene-environment example and parts of the discussion. All authors participated substantially in the writing of the submitted manuscript and approved the submitted version.

## Endnotes

### Appendix 1: Computation of expected numbers for a given design and scenario

Let Z denote the proxy variable for X, the exposure of interest and define

- $\tau_{j}^{0}$ the Phase One proportion of the j^th ^stratum within controls.

- $\pi_{jk}^{0}$ the Phase Two proportion of the k^th ^outcome of X within stratum j of controls.

The proportion of cases in each stratum depends on the corresponding proportion of controls and the assumed odds ratios (*ψ*_*k*_). Let us denote by

q_j _the stratum-specific weighted odds ratio, $q_{j}=\sum_{k}\pi_{jk}^{0}\times \psi_{k}$

$\tau_{j}^{1}$ the Phase One proportion of the j^th ^stratum within cases, $\tau_{j}^{1}=\frac{\tau_{j}^{0}\times q_{j}}{\sum_{j}\tau_{j}^{0}\times q_{j}}$

$\pi_{jk}^{1}$ the Phase Two proportion of the k^th ^outcome of X within stratum j of cases, $\pi_{jk}^{1}=\pi_{jk}^{0}\times \frac{\psi_{k}}{q_{j}}$

The flexible two-phase approach starts with fixed numbers of controls (n_0*j*_) and cases (n_1*j*_), from which one computes

- *N*_*ij*_, the expected Phase One numbers of cases and controls to be screened in each stratum j,

- *n*^*i*^_*jk*_, the expected Phase Two stratum-wise numbers in each exposure category k

Phase One:

The overall expected number of Phase One controls *N*_*0 *_and cases *N*_*1 *_to be screened are $N_{0}=\max\left(\frac{n_{0j}}{\tau_{j}^{0}}\right)$ and $N_{1}=\max\left(\frac{n_{1j}}{\tau_{j}^{1}}\right)$

From these, one obtains the stratum-specific expected Phase One numbers

*N*_0*j *_= *N*_0 _× $\tau_{j}^{0}$ and *N*_1*j *_= *N*_1 _× $\tau_{j}^{1}$

Phase Two:

The expected numbers in each Phase Two exposure category are computed as $n_{jk}^{0}=n_{0j}\times \pi_{jk}^{0}$ and $n_{jk}^{1}=n_{1j}\times \pi_{jk}^{1}$

### Appendix 2: Expected numbers for Example 1

Using the notations from appendix 1, let J = 2 and K = 2,

*τ*^*0*^_*j *_(the Phase One proportions), $\pi_{jk}^{0}$ (the stratum-wise Phase Two proportions) among controls and *ψ*_2 _the odds ratio with MWF exposure take the values presented in Table 1.

Then, following the formula given in appendix 1,

the weighted odds-ratio in stratum 1 of non metal-workers is $q_{1}=\pi_{11}^{0}\times \psi_{1}+\pi_{12}^{0}\times \psi_{2}=0.975\times 1+0.025\times 2=1.025$

the weighted odds-ratio in stratum 2 of metal-workers is $q_{2}=\pi_{21}^{0}\times \psi_{1}+\pi_{22}^{0}\times \psi_{2}=0.75\times 1+0.25\times 2=1.25$

From this, we obtain the Phase Two proportions of metal-fluid exposure (k = 2);

In stratum 1 of non-metal working cases: $\pi_{12}^{1}=\pi_{12}^{0}\times \frac{\psi_{1}}{q_{1}}=0.025\times \frac{2}{1.025}=4.9\%$

In stratum 2 of metal-working cases: $\pi_{22}^{1}=\pi_{22}^{0}\times \frac{\psi_{2}}{q_{2}}=0.25\times \frac{2}{1.25}=40\%$

The Phase One proportion of metal-workers among cases is $\tau_{2}^{1}=\frac{\tau_{2}^{0}\times q_{2}}{\sum_{j}\tau_{j}^{0}\times q_{j}}=\frac{0.20\times 1.25}{0.20\times 1.25+0.80\times 1.025}=0.234$

From these quantities, the expected numbers can be derived for a given design as illustrated in table 2

## Supplementary Material

Additional file 1 This is a text file containing the code of the Stata statistical software (StataCorp. 2007; Stata Statistical Software: Release 9 and onwards. College Station, TX: StataCorp LP.) for fitting two-phase data. It can be accessed using any text processor but can only be executed within Stata. It should be saved under the name blogit_2P.ado. Click here for file

Additional file 2 This is a help file describing the preceding program and its options. In can only be displayed as a help file from within Stata. It should be saved under the name blogit_2P.hlp. Click here for file

Additional file 3 This is a text file containing the code of the Stata statistical software for performing the power computation for Example 1 using the above program and performing the computations shown in figure 1. In reads in the data in the data file MWF.raw included as Additional file 4. It can be accessed using any text processor but can only be executed within Stata. It should be saved under the name figure1.do. Click here for file

Additional file 4 This is text file containing the data obtained by performing the computations shown in Appendix 2. It is used by the Stata program figure1.do included as Additional file 3. It should be saved under the name mwf.raw. Click here for file
